# A coconut oil-rich meal does not enhance thermogenesis compared to corn oil in a randomized trial in obese adolescents

**Published:** 2017-04-17

**Authors:** Janna LaBarrie, Marie-Pierre St-Onge

**Affiliations:** Institute of Human Nutrition (JL, MPSO) and Department of Medicine (MPSO), Columbia University Medical Center, New York, USA

**Keywords:** Triglycerides, Thermogenesis, Weight management, Obesity, Cholesterol, Blood pressure

## Abstract

**Background:**

Consumption of medium chain triglycerides (MCT) in overweight adults increases thermogenesis and improves weight management. Coconut oil is a rich natural source of MCT, but its thermogenic effect is unknown. Our study evaluated the effects of a test oil enriched in coconut oil, on energy expenditure, satiety, and metabolic markers in a randomized, double blind, cross-over study.

**Methods and findings:**

Fifteen children, age 13-18 years, body mass index >85th percentile for age and sex, were enrolled. Two test meals, containing 20 g of fat from either corn oil or a coconut oil-enriched baking fat (1.1 g of fatty acids with chain lengths ≤ 10C), were administered. A fasting blood sample was taken before breakfast and at 30, 45, 60, 120, and 180 min post-meal for measurement of metabolites. Thermic effect of food (TEF) was assessed over 6 h using indirect calorimetry. Satiety was measured using visual analog scales (VAS). There was no significant effect of fat type, time, or fat type × time interaction on TEF, appetite/satiety, glucose, and insulin area under the curve. There was a significant effect of fat type on leptin (P=0.027), triglycerides (P=0.020) and peptide YY (P=0.0085); leptin and triglyceride concentrations were lower and peptide YY concentrations were higher with corn oil consumption.

**Conclusion:**

A coconut oil-enriched baking fat does not enhance thermogenesis and satiety in children. Given that this is the only current study of its kind, more research is needed into the use of coconut oil as a tool in weight management in overweight and obese children.

## Small Dose MCFA and Thermic Effect of Food

### Introduction

In the United States, approximately 17% of children aged 2-19 years are obese and 31.8% are either overweight or obese [1,2]. Although the increase in childhood obesity observed over the past 30 y has slowed recently, the prevalence remains high even on a global scale, resulting in high economic burden [3]. Children with over weight and obesity are at increased risk for dyslipidemia and abnormal levels of cardio-metabolic risk factors; high blood pressure, cholesterol and triglycerides due to obesity are associated with adverse cardiovascular health outcomes [4-6]. There is strong evidence that obesity and cardio metabolic factors developed in childhood track into adulthood [7,8] making childhood obesity a top area of public health concern and a target for current research efforts.

Current traditional interventions for overweight children concentrate on treating the disease and improving health outcomes through lifestyle changes. Overall, the research on the effectiveness of interventions involving a dietary component alone among children suffering from overweight and obesity is lacking and conflicting; more work is needed to determine the benefits of specific dietary components in weight management interventions [9].

Studies have shown that diet can influence metabolism and thermogenesis in adults. Medium chain triglycerides, containing fatty acids with 6-10 carbon atoms, have been proposed as agents that could assist with weight management [10]. In a previous study, MCT oil consumption resulted in lower food intake than consumption of a fat rich in long chain triglycerides in overweight men [11]; similarly, another showed that MCT oil supplementation decreased food intake in 12 healthy adult males [12]. In addition to effects on satiety and food intake, MCT may also enhance energy expenditure and weight loss. St-Onge et al. showed that consumption of MCT over approximately 1 month increased energy expenditure and fat oxidation in women [13] and men [14] and that consumption of 18-24 g MCT oil over 16 weeks enhanced weight loss in overweight adults [15]. Taken together, these studies show that there may be a role for the use of MCT oil as a tool in weight management in adults with overweight and obesity.

While evidence in adults shows that fats containing MCTs may be preferable to those containing long chain triglycerides for weight management, no study has investigated the effects of MCT on energy metabolism in children. However, purified MCT oil is not readily available and expensive commercially. Coconut oil is the best source of naturally-occurring MCT, with over 12% of fatty acids from MCT and close to 55%, if including lauric acid [16], which some consider a medium chain fatty acid [17-19]. Therefore, studying the effects of a coconut oil-rich meal may have relevance for public health. One study conducted in Brazil showed reductions in waist circumference in a group of women consuming coconut oil, but not in the control group consuming soybean oil [20]; however, group differences were not statistically compared. In order to explore the role of coconut oil as a tool in weight management in overweight and obese children, our study seeks to determine the effects of a baking fat enriched in coconut oil on postprandial energy expenditure, satiety and metabolic markers in adolescents. This study was responsive to a call for applications on childhood obesity by the USDA for its Small Business Innovation Research program to stimulate innovative research in the private sector and promote partnerships with academic researchers. We hypothesized that 20 g of fat rich in coconut oil would: 1) enhance the thermic effect of food (TEF) and improve satiety, and 2) lower levels of glucose, insulin, triglycerides, leptin, and peptide YY (PYY) compared to 20 g of corn oil. These metabolites were chosen since they had previously been found to be influenced by MCT consumption in adults [11].

## Subjects and Methods

This study employed a 2 arm, double-blind, randomized crossover design. A recruitment goal of 30 boys and girls, age age 13-18 y, with BMI>85th percentile, was set, with an expected final sample size of 25. Based on pilot data we calculated having 80% power to detect a difference of 13.3 kcal × 5 h in TEF AUC after the breakfast meal with 95% confidence, assuming a within subject standard deviation of 22.8 kcal. The study protocol was approved by the Institutional Review Boards of St. Luke's/Roosevelt Hospital Center and Columbia University Medical Center (New York, NY). All participants provided informed consent prior to the start of the study. The study was conducted in 2 separate phases (studies) registered on clinicaltrials.gov (NCT#02132377 and NCT#02346994). In phase 1, the coconut oil-enriched baking fat was developed and tested in a small, proof of concept clinical study. In phase 2, the larger clinical study was performed. Data from both phases are presented herein. A total of 27 adolescents gave informed consent to participate in this study and 15 participated in at least one test day. Of those, 4 participants were enrolled in study 1 and 11 participants were enrolled in study 2. One participant failed to complete both test days.

Recruitment entailed an initial phone screening with interested teenagers or their parent/guardian to determine eligibility and explain the study. If potentially eligible, a second, in-person screening was scheduled. All participants were required to be weight stable (± 2.5 kg for at least 3 months prior to screening). Exclusion criteria included the presence of any endocrine disorder, attempts to lose weight in the past 3 months, use of medication, asthma, anemia, psychosis, bipolar disorder, major depression, autism, behavioural disorders, use of investigational medications or devices, allergies to foods included in the study, and use of over the counter supplements that may affect the study endpoints. All participants were given the opportunity to ask questions about the study prior to signing assent or informed consent.

Eligible participants were randomized into the study using a random digit generator, and scheduled for their first test day. The study included two test meals, each providing 20 g of fat from either the coconut oil-enriched baking fat or corn oil (Table 1). Test days were separated by a wash out period of 2-4 weeks. Girls were tested at 4 weeks intervals to ensure that testing occurred in the same phase of their menstrual cycle.

The coconut oil enriched baking fat contained predominantly lauric (C12:0), palmitic (16:0) and oleic (18:1) acids and was provided by Prosperity Organic Foods, Inc. (Boise, ID). Caprylic and capric acids together contributed approximately 5% of total fatty acids. Test oils were incorporated in muffins prepared from an apple spice muffin mix (Bob's Red Mills, Milwaukie, OR) and stored in the Bionutrition Unit at the Columbia University Irving Institute for Clinical and Translational research. Staff of the Bionutrition Unit assigned a color code to the muffins to blind participants and investigators to the oil assignment. The muffin contained 450 kcal and 20 g of fat from either the coconut oil-enriched baking fat or corn oil. Test muffins contained approximately 1.1 g of caprylic and capric acids (MCT). Participant energy requirements were calculated using the Schofield equation [21] as follows according to sex, height (H) and weight (W) for ages 10-18 years:

**Males:** (16.25 × W (kg)) + (137.2 × H (m)) + 515.5

**Females:** (8.365 × W (kg)) + (465 × H (m)) + 200

Breakfast provided 35% of resting energy requirements. If needed, in addition to the muffin, vanilla-flavored yogurt (150 kcal, LaYogurt Probiotic, Flemington, NJ) and sweetened applesauce were added to reach the target calorie level.

On the day prior to testing, participants were asked to refrain from performing any structured exercise and to record their food intake that day (the same foods were asked to be consumed the day before the second test day). On test day, participants were asked to come to the research center at 7:30 AM after a 12 h fast starting at 8:00 PM the night before. Upon arrival, participants' height and weight were measured. A catheter was then inserted in an upper arm vein. Participants rested for 30 min, after which resting energy expenditure (REE) was measured for 45 min using an indirect calorimetry cart (Vmax29, Sensor Medics, Yorba Linda, CA). A fasting blood sample was obtained following the REE measurement. The participant consumed their breakfast within 10 min following the first blood sample. Immediately after breakfast consumption, another blood sample was obtained (time 0). Additional blood samples were taken at 30, 45, 60, 120 and 180 min after breakfast. REE measurement was performed for 35-40 min of each hour over a period of 6 h following breakfast consumption. Participants were given 15-20 min breaks every hour. Data from the first and last 5 min of each hourly measurement period were discarded as those represent time to achieve steady-state (fist 5 min) or increased restlessness (last 5 min). The remaining 30 min were averaged for each hour. TEF was calculated by subtracting the averaged REE from averaged postprandial energy expenditure data for each time period. TEF area under the curve (AUC) was calculated using the trapezoidal method.

During the 15-20 min hourly breaks, participants were asked to complete visual analog scales (VAS) to report their feelings of appetite and satiety. VAS scales were given in the fasted state, before and after muffin consumption, and every hour until the end of the testing period.

**Questions on the VAS included:** 1) How hungry do you feel? 2) How satisfied do you feel? 3) How full do you feel? 4) How much do you think you could eat? 5) How sluggish do you feel? 6) How energetic do you feel? at that moment in time. Participants were asked to place a mark on a 100-mm line anchored with “not at all” (0) and “extremely” (100).

Blood samples were only available for the 11 children who participated in phase 2 (one failed to complete test day 2). Once drawn, samples were placed on ice, processed immediately and stored in -80°C freezers until analysis. Samples were analyzed for glucose, insulin, triglycerides, PYY and leptin as done previously [11].

Data were analyzed using linear mixed model analysis, with ID as random variable, and with a number of different outcomes. Baking fat type was used as the main predictor variable; wherever available, time was used as an independent variable, as was its interaction with baking fat type. For the VAS data, fasting time was coded as “-1”, when used as an independent variable; testing day (1 vs. 2) was also used as an independent variable in every analysis. Sex, age, body weight and race were used as covariates. Baking fat type (coconut oil-enriched fat or corn oil) was coded for blinding. The code was revealed after the completion of all statistical analyses. REE and VAS data were analyzed for all participants (n=15); metabolites were analyzed for completers in phase 2 only (n=10). Data are reported as means ± SD. Significance was established as P<0.05.

## Results

The final sample consisted of 14 completers and 1 participant who completed 1 of 2 test days and voluntarily dropped out of the study before test day 2. Of those participants, 4 were enrolled in phase 1. Data from all 15 participants were included in the final analysis. Phase 1 included 1 male and 3 females, age 16 ± 1.4 years and BMI 30.7 ± 5.2 kg/m^2^. Phase 2 included 6 males and 5 females, age 16.8 ± 1.4 y and BMI 32.3 ± 4.1 kg/m^2^. Males were significantly older than females (P=0.006) and consumed more calories for breakfast (P<0.001). Height, weight, and BMI were comparable (as shown in Table 2).

### Energy expenditure measurements

**Figure 1:**
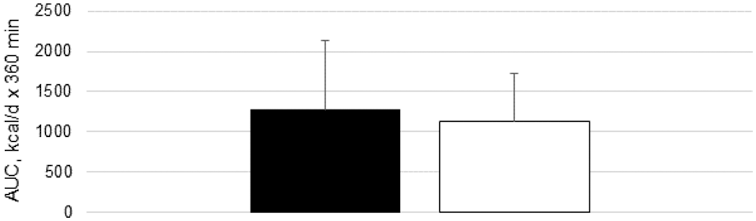
Thermic effect of food AUC over 6 h following consumption of test breakfasts containing 20 g of fat from corn oil (black bar) or an MCT-enriched baking fat (white bar), Data are means ± SD, n=15.

There was no significant effect of fat type (P=0.72), time (P=0.54) or fat type × time interaction (P=0.78) on REE (Table 3). Similarly, the TEF was not affected by fat type (P=0.58), time (P=0.54) or fat type × time interaction (P=0.75). The AUC for REE and TEF (as shown in Figure 1) were not different between test fats (P=0.90 and 0.31, respectively). Respiratory quotient was not affected by fat type (P=0.39), time (P=0.76) or fat type × time interaction (P=0.63).

### Satiety

**Figure 2:**
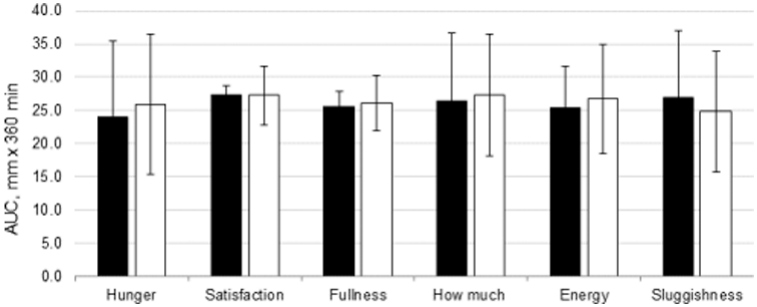
AUC for each VAS question over 6 h following consumption of test breakfasts containing 20 g of fat from corn oil (black bars) or an MCT-enriched baking fat (white bars). Data are means ± SD, n=15.

Data from the VAS did not reveal any effect of fat type on hunger (P=0.82), satiety (P=0.55), prospective food intake (P=0.92) and energy level (P=0.91) (as in Figure 2). However, participants rated greater sluggishness overall with corn oil consumption (P=0.034). There was no fat type × time interaction on any of the VAS measures (all P>0.27).

### Hormone variables

There was no significant effect of fat type (P>0.69), time (P>0.28) or fat type × time interaction (P>0.50) on glucose and insulin concentrations. There was a significant effect of fat type on leptin (P=0.027), triglycerides (P=0.020) and PYY (P=0.0085). For these variables, leptin and triglyceride concentrations were lower with corn oil consumption and PYY concentrations were higher. The effect of time was only significant for triglyceride concentrations (P<0.001) and there were no fat type × time interactions on any of the measures (all P>0.27).

## Discussion

The results of this study show that a coconut oil-enriched baking fat does not affect REE, TEF, respiratory quotient, satiety, glucose and insulin compared to the same amount of corn oil in overweight and obese children. However, some satiety signals were different between testing conditions (leptin and PYY) but the effects were in opposite directions than those expected. In addition, the lack of fat type × time interaction shows that the effect may not be due to consumption of the test fat and definite conclusions about these findings cannot be reached at this time. Although we did not detect any differences between the two fat types, this study is the first to investigate the effects of high coconut oil consumption in children and may serve as a starting point for further exploration into the use of designer fats for childhood overweight and obesity.

We expected to find increased TEF with acute consumption of the coconut oil-enriched meal relative to the corn oil-enriched meal. White et al. [22] provided young, normal weight adult females a test diet with similar content of fats with fatty acid chain lengths ≤ 12 C (approximately 8% of caprylic + capric acids and 18% of lauric acid) and compared REE after 7 and 14 days of consumption to a diet rich in saturated fats from beef tallow. Postprandial EE was greater with higher medium chain fat diet consumption compared to the beef tallow-rich diet. Differences in postprandial EE between diets were attenuated after 14 days of consumption. Fat oxidation rates were also greater at 7 days, but not 14 days, with MCT consumption compared to beef tallow.

In subsequent studies, we proposed that larger MCT doses, particularly from capric and caprylic acids, may be necessary for sustained enhancement of postprandial thermogenesis. The current study results support this view. In our earlier investigations, we provided purified MCT oil in diets for periods of 4 to 16 weeks [13-15]. Participants consumed at least 18 g of caprylic + capric acids daily. Dulloo et al. randomized men to receive various proportions of MCT and long chain triglycerides up to 30 g and showed that EE increased in a dose response manner with increasing MCT treatment, with the lowest significant increase in 24 h EE of 5% seen with intakes of 15 g [23]. In the current study, test muffins provided approximately 1 g of caprylic (C8:0) and capric (C10:0) acids, which were the fatty acids provided in all previous research [11-15,23]; including lauric acid (C12:0) brings the total to 4.7 g of MCFA. Research by Kasai et al. showed that as little as 5-10 g of MCT oil, provided in a meal, raised TEF in Japanese adults relative to long chain triglycerides from rapeseed and soybean oil [21]. However, according to findings by Dulloo et al. [23], the amount of caprylic and capric acids provided in 20 g of our MCT-enriched fat may not have been sufficient to stimulate EE. Our muffins contained 20 g of fat, 40% of their energy content. This amount was chosen as one that would be reasonable to include in a single food. In order to achieve 15 g of caprylic and capric acids from coconut oil would have required over 300 g of fat. If one considers that lauric acid contributes to the EE-enhancing effects of MCT, then a lesser amount of coconut oil would have been needed to reach 15 g of MCT, approximately 63 g. This is still much too high for a single food item. Therefore, it is very likely that the small quantity of caprylic and capric acids provided in our test meal was insufficient to raise postprandial EE above that exerted by the control oil and that lauric acid was not an important contributor to this thermogenic effect.

Another potential explanation for the lack of effect of our test oil on TEF may be exposure duration, which may be particularly relevant at such low doses. We previously observed significant increases for MCT compared to long chain saturated fat enriched diet in both fat oxidation and EE in 17 overweight women after 27 days of consumption (13). As an example, we have shown that, while no acute differences were observed at a first test meal, milk supplementation enhanced the TEF and EE in children relative to a single nutrient beverage after 6 days of continued exposure to the high milk intake [24]. Perhaps continued intake of the coconut oil enriched fat over time would stimulate an increase in TEF in adolescents. This hypothesis would need to be tested.

We expected our coconut oil enriched baking fat to enhance satiety and reduce appetite. This was not observed. St-Onge et al. [11] showed that food intake at lunch was reduced following a preload containing 10 g MCT oil (caprylic + capric acids) in overweight men compared to corn oil. Another study in 12 healthy men showed that among breakfasts containing olive oil, lard, fat substitute or MCT, food intake was lowest in the MCT [12]. Contrary to these findings, we did not see differences in any of the subjective measures of appetite and satiety between the 2 test meals. We did not however, compare prospective food intake after the two test meals or the effect of a preload on satiety after breakfast consumption. Measuring satiety using VAS can be difficult due to subjective variability, especially among adolescents.

Exactly how coconut oil may influence appetite is not clear. In the present study, we investigated levels of various hormones and metabolites after consumption of the 2 test meals. We did not find any difference in levels of glucose and insulin but triglycerides were lower, leptin and PYY higher with corn oil consumption. These results contradict prior data obtained by our group [11] showing a lower rise in insulin and triglycerides and greater rise in PYY after MCT oil consumption compared to corn oil provided in a breakfast meal that contained 20 g of fat. The difference observed in these hormones and metabolites could be a result of the higher amount of caprylic + capric acids provided (20 g vs. 1 g), once again highlighting the possibility that there is a minimum threshold of MCT needed to produce an effect.

Our study had several limitations. First, our MCT enriched fat included primarily lauric acid from coconut oil. All other studies finding enhanced TEF and fat oxidation from MCT consumption have been done with MCT containing caprylic and capric acids [11-15,23]. However, based on research by DeLany et al. [25], one could expect lauric acid to enhance fat oxidation relative to long chain fatty acids. Other limitations include the small amount of MCT included in the test muffin and short term nature of the study, as discussed above. Finally, our sample size was limited due to recruitment challenges.

## Conclusion

The results of our study suggest that small doses of MCT do not affect EE, satiety or insulin levels in overweight and obese adolescents. However, given that this is the first study to investigate the effects of MCT on these outcomes in children, we believe that more research is needed in order to fairly evaluate the use of MCT as a tool in weight management for childhood obesity. Initial studies using refined MCT oil, composed of 100% caprylic and capric acids should be undertaken. Using this study as a resource, future research should begin to investigate the metabolic effects of consuming larger amounts of MCT, within a reasonable dose range, from natural sources such as coconut oil and palm kernel oil, for longer duration in both adults and children.

## Figures and Tables

**Table 1:** Fatty acid composition of the MCT-enriched baking fat. Data represent the percent of total fat from each fatty acid in the baking fat and test muffin. One serving of the baking fat contains 9 g of total fat.

Fatty acid	Coconut oil enriched baking fat	Amount in test muffin (g)	Corn oil^1^
SFA:PUFA:MUFA	52:06:42	10.4:1.2:8.4	13.4:54:28.1
Butyric acid, %	0.2 ± 0	0.04	
Caproic acid, %	0.27 ± 0.14	0.054	
Caprylic acid, %	2.66 ± 0.96	0.53	
Capric acid, %	2.67 ± 0.92	0.53	
Lauric acid, %	18.02 ± 2.2	3.6	
Myristic acid, %	6.78 ± 1.61	1.36	
Palmitic acid, %	17.6 ± 2.28	3.52	11.5
Palmitoleic acid, %	0.33 ± 0.28	0.066	0.1
Stearic acid, %	4.15 ± 1.59	0.83	1.74
Oleic acid, %	40.73 ± 4.13	8.15	29
Linoleic acid, %	6.37 ± 3.13	1.27	55.6
Alpha-Linolenic acid, %	0.21 ± 0.14	0.042	0.95
Arachidonic acid, %	0.19 ± 0.1	0.038	0.4
Eicosenoic acid, %	0.09 ± 0.06	0.018	0.27
MCFA (C8-10), g/muffin	1.06		0

Muffins included 20 g of fat from the baking fat,

1 Information obtained from Maki et al. [26].

**Table 2:** Characteristics of study participants.

	Males (n=7)	Females (n=8)
Age, y	17.6 ± 0.8	15.8 ± 1.3
Height, cm	174.6 ± 11.0	169.3 ± 6.6
Weight, kg	94.4 ± 15.3	94.1 ± 16.9
BMI, kg/m^2^	30.9 ± 3.2	32.7 ± 5.1
Race/ethnicity		
Hispanic/Black	1	1
Hispanic/Multiple race	4	2
Non-Hispanic/Black	1	5
Non-Hispanic/White	1	0
Average intake at breakfast, kcal	799 ± 86	622 ± 55

**Table 3:** Mean outcomes for each test oil. Data are raw means ± SD, n=14, for the 6 h postprandial period (resting energy expenditure, thermic effect of food and respiratory quotient) or 3 h sampling period (glucose, insulin, triglycerides, leptin, peptide YY). Blood samples were analyzed for completers of phase 2 only, n=10.

Outcome variable	MCT-enriched baking fat	Corn oil
Resting energy expenditure, kcal/d	1958 ± 312	1930 ± 299
Thermic effect of food, kcal/d	235 ± 129	252 ± 188
Respiratory quotient	0.888 ± 0.023	0.0873 ± 0.041
Glucose, mg/dL	105.7 ± 29.3	108.3 ± 26.8
Insulin, uU/mL	73.8 ± 31.0	68.1 ± 35.6
Triglycerides, mg/dL	77.1 ± 26.3*	71.9 ± 23.1
Leptin, ng/mL	16.5 ± 13.0*	17.2 ± 14.7
Peptide YY, pg/dL	61.6 ± 14.0*	67.3 ± 17.2

One participant did not have sufficient samples for PYY analyses,

* Significant effect of fat type, P<0.05.

## Abbreviations

BMI: Body Mass Index
EE: Energy Expenditure
MCFA: Medium Chain Fatty Acids
MCT: Medium Chain Triglycerides
REE: Resting Energy Expenditure
RMR: Resting Metabolic Rate
RQ: Respiratory Quotient
TEF: Thermic Effect Of Food
VAS: Visual Analog Scales
